# Supplementary material for: Insights into opium poppy (Papaver spp.) genetic diversity from genotyping-by-sequencing analysis
Source: Sci Rep. 2022 Jan 7;12:111. doi: 10.1038/s41598-021-04056-3 (PMC8741915; doi:10.1038/s41598-021-04056-3)
Supplement: Supplementary file 1 — Supplementary Information. [file 41598_2021_4056_MOESM1_ESM.pdf]

Supporting Information

Insights into opium poppy (*Papaver* spp.) genetic diversity from genotyping-by-sequencing analysis

Uyen Vu Thuy Hong[1,2], Muluneh Tamiru-Oli[1,2,*], Bhavna Hurgobin[1,2], Christopher R. Okey[3], Artur R. Abreu[3], Mathew G. Lewsey[1,2,*]

[1]La Trobe Institute for Agriculture and Food, La Trobe University, AgriBio Building, Bundoora, VIC 3086, Australia

[2]Australian Research Council Research Hub for Medicinal Agriculture, La Trobe University, AgriBio Building, Bundoora, VIC 3086, Australia

[3]Palla Pharma Ltd, Docklands, VIC 3008, Australia.

Table S1. List of the *Papaver* accessions include in this study. All accessions were sourced from the poppy germplasm collections at the Leibniz Institute of Plant Genetics and Crop Plant Research (IPK), Germany.

No.	Acc ID	Species	Origin	Common name
1	PAP 8	*P. somniferum*	Turkey	
2	PAP 12	*P. somniferum*	France	Tourneur-Freres-Typ D
3	PAP 28	*P. somniferum*	Yugoslavia (former)	
4	PAP 56	*P. somniferum*	Germany	
5	PAP 59	*P. somniferum*	Turkey	
6	PAP 71	*P. somniferum*	Hungary	Pulawski Niebieski
7	PAP 77	*P. somniferum*	Hungary	Pitvarositf
8	PAP 78	*P. somniferum*	Hungary	Fertodi Soktoku Kek
9	PAP 90	*P. somniferum*	Bulgaria	Voronezskij 1042
10	PAP 107	*P. somniferum*	France	Flora II
11	PAP 133	*P. somniferum*	Czechoslovakia	Erbachshofsky
12	PAP 140	*P. somniferum*	Bulgaria	
13	PAP 142	*P. somniferum*	Turkey	
14	PAP 146	*P. somniferum*	Russia	Altaec
15	PAP 149	*P. somniferum*	Morocco	violet plat
16	PAP 150	*P. somniferum*	Morocco	coralla v. majore
17	PAP 151	*P. somniferum*	Morocco	blanc large
18	PAP 152[a]	*P. somniferum*	Morocco	blanc carre
19	PAP 156	*P. somniferum*	Bulgaria	Tatarstan I
20	PAP 160	*P. somniferum*	Yugoslavia (former)	
21	PAP 165	*P. somniferum*	Bulgaria	Fertodi Zarttoku Kek
22	PAP 167	*P. somniferum*	China	
23	PAP 197	*P. somniferum*	Czechoslovakia	Susicky Cervenosemenny
24	PAP 200	*P. somniferum*	Japan	Ikkanshu
25	PAP 201	*P. somniferum*	Germany	Daubaer Silbergrauer
26	PAP 238	*P. somniferum*	Czechoslovakia	Mahndorfer
27	PAP 246	*P. somniferum*	Ukraine	Lubenskij 6
28	PAP 255	*P. setigerum*	Portugal	
29	PAP 271	*P. somniferum*	Slovakia	
30	PAP 281	*P. somniferum*	Slovakia	
31	PAP 294	*P. somniferum*	Slovakia	
32	PAP 299	*P. somniferum*	Slovakia	
33	PAP 327	*P. somniferum*	Bulgaria	Sinjocaftjast P 360
34	PAP 333	*P. somniferum*	Germany	
35	PAP 335	*P. somniferum*	Poland	
36	PAP 336	*P. somniferum*	Switzerland	
37	PAP 337	*P. somniferum*	Switzerland	
38	PAP 354	*P. somniferum*	Italy	
39	PAP 393	*P. somniferum*	Slovakia	
40	PAP 400[b]	*P. somniferum*	Spain	
41	PAP 452	*P. somniferum*	United Kingdom	Pink Chiffan
42	PAP 466	*P. somniferum*	Russia	Daneborg
43	PAP 469[b]	*P. somniferum*	Germany	
44	PAP 478	*P. somniferum*	Poland	
45	PAP 483	*P. somniferum*	Poland	
46	PAP 496	*P. somniferum*	Germany	
47	PAP 499	*P. somniferum*	France	

48	PAP 518	*P. somniferum*	Belgium	
49	PAP 599	*P. somniferum*	Czechoslovakia	Hatvani
50	PAP 612	*P. somniferum*	Czechoslovakia	Selecty Stribrosedy
51	PAP 630	*P. somniferum*	Vietnam	
52	PAP 683[b]	*P. somniferum*	Italy	
53	PAP 692	*P. somniferum*	Poland	
54	PAP 696	*P. somniferum*	Spain	
55	PAP 715	*P. somniferum*	Belgium	
56	PAP 719	*P. somniferum*	Kazakhstan	
57	PAP 720	*P. somniferum*	Russia	
58	PAP 727	*P. somniferum*	Austria	
59	PAP 733	*P. somniferum*	Vietnam	
60	PAP 739	*P. somniferum*	Italy	
61	PAP 747[a]	*P. somniferum*	Portugal	
62	PAP 749	*P. somniferum*	Portugal	
63	PAP 761	*P. somniferum*	Japan	Ikkan
64	PAP 764	*P. somniferum*	North Korea	
65	PAP 765	*P. somniferum*	North Korea	
66	PAP 766	*P. somniferum*	North Korea	
67	PAP 767	*P. somniferum*	North Korea	
68	PAP 768[a]	*P. somniferum*	North Korea	
69	PAP 773	*P. somniferum*	Denmark	Parmo
70	PAP 777	*P. somniferum*	Germany	
71	PAP 784	*P. somniferum*	Romania	
72	PAP 790	*P. somniferum*	Turkey	
73	PAP 792	*P. somniferum*	Romania	
74	PAP 795	*P. somniferum*	Romania	
75	PAP 796[b]	*P. setigerum*	Italy	
76	PAP 815	*P. somniferum*	China	
77	PAP 814	*P. somniferum*	Romania	
78	PAP 816	*P. somniferum*	USA	Pink Single Poppy
79	PAP 823[b]	*P. somniferum*	Romania	mac
80	PAP 827[a]	*P. somniferum*	Croatia	mak (kroatischer Name fur Mohn)
81	PAP 830	*P. somniferum*	Croatia	mak
82	PAP 835	*P. somniferum*	Germany	
83	PAP 845	*P. somniferum*	Croatia	
84	PAP 932	*P. somniferum*	Hungary	KASSA
85	PAP 1025	*P. somniferum*	Austria	
86	PAP 1062	*P. somniferum*	Austria	
87	PAP 1090	*P. somniferum*	Iran	MOHN AUS PERSIEN II
88	PAP 1129	*P. somniferum*	Denmark	LORI
89	PAP 1142	*P. somniferum*	Iran	MOHN AUS PERSIEN
90	PAP 1168	*P. somniferum*	Turkey	NYAZI MUTAFT
91	PAP 1185	*P. somniferum*	Russia	VILAR-2
92	PAP 1224	*P. somniferum*	Russia	MAJAK
93	PAP 180 A	*P. somniferum*	Mongolia	
94	PAP 180 B	*P. somniferum*	Mongolia	
95	PAP 229 A	*P. setigerum*	Spain	

[a]Accessions not included for GBS analysis.

[b]Accessions not included for alkaloid profiling.

Table S2. Summary statistical of data generated from GBS of 91 *Papaver* accessions.

**Steps**	**Statistics**	**Total size (Gb)**
**Data generation and alignment**		
Raw reads	103,802,122	15.57
Average number of raw reads per sample	1,140,683	
Number of low quality (<10) reads removed	89,099	
Number of "no Radtag" removed	111,744	
Number of filtered reads retained	103,601,279	15.54
Average number of filtered read per sample	1,134,476	
Average mapping rate per sample	99.78%	
**SNP calling**		
Total loci	601,567	
Retained loci	76,407	
Total SNPs	165,363	

Table S3. Summary of total raw GBS reads generated, reads retained after filtering and alignment rate for all the 91 *Papaver* accessions sequenced.

No	Accession ID	Raw reads	Filtered reads	Alignment rate (%)
1	PAP 8	1,372,600	1,368,192	99.83
2	PAP 12	1,121,416	1,119,700	99.87
3	PAP 28	1,281,905	1,280,107	99.87
4	PAP 56	1,397,053	1,394,964	99.86
5	PAP 59	1,282,005	1,280,480	99.85
6	PAP 71	914,503	913,099	99.87
7	PAP 77	1,419,364	1,414,747	99.87
8	PAP 78	,661,836	1,659,405	99.89
9	PAP 90	1,451,089	1,449,191	99.89
10	PAP 107	1,570,650	1,568,447	99.88
11	PAP 133	1,392,292	1,390,512	99.88
12	PAP 140	1,270,996	1,265,976	99.71
13	PAP 142	1,367,621	1,363,011	99.83
14	PAP 146	1,109,457	1,107,676	99.87
15	PAP 149	1,042,857	1,041,270	99.81
16	PAP 150	1,247,034	1,245,198	99.82
17	PAP 151	967,586	966,278	99.82
18	PAP 156	1,109,777	1,108,345	99.87
19	PAP 160	1,512,681	1,507,223	99.86
20	PAP 165	1,435,248	1,433,276	99.84
21	PAP 167	1,580,845	1,578,329	99.86
22	PAP 197	1,411,310	1,409,104	99.88
23	PAP 200	1,195,684	1,193,613	99.83
24	PAP 201	1,291,436	1,289,841	99.88
25	PAP 238	1,089,293	1,086,422	99.88
26	PAP 246	1,056,573	1,055,252	99.87
27	PAP 255	1,188,660	1,186,821	97.31
28	PAP 271	810,856	809,801	99.87
29	PAP 281	946,870	945,700	99.87
30	PAP 294	1,031,744	1,030,481	99.89
31	PAP 299	1,594,481	1,590,483	99.89
32	PAP 327	1,289,790	1,288,211	99.85
33	PAP 333	1,350,891	1,348,766	99.87
34	PAP 335	1,358,850	1,357,091	99.89
35	PAP 336	1,285,253	1,283,741	99.84
36	PAP 337	1,334,204	1,332,525	99.84
37	PAP 354	1,241,611	1,239,498	99.83
38	PAP 393	987,723	986,496	99.88
39	PAP 400	985,611	983,763	99.68
40	PAP 452	1,093,853	1,091,522	99.85
41	PAP 466	981,975	980,759	99.86
42	PAP 469	1,035,352	1,034,091	99.85
43	PAP 478	1,166,478	1,164,390	99.87
44	PAP 483	1,237,596	1,235,857	99.88
45	PAP 496	1,185,615	1,183,879	99.86
46	PAP 499	1,199,747	1,197,206	99.88
47	PAP 518	1,058,117	1,056,776	99.84
48	PAP 599	1,165,399	1,163,971	99.89

49	PAP 612	1,039,595	1,030,058	99.89
50	PAP 630	1,238,549	1,236,340	99.83
51	PAP 683	1,067,715	1,066,161	99.86
52	PAP 692	847,072	845,900	99.87
53	PAP 696	1,009,504	1,007,944	99.83
54	PAP 715	1,271,977	1,270,102	99.87
55	PAP 719	1,286,815	1,275,048	99.88
56	PAP 720	1,310,790	1,308,355	99.87
57	PAP 727	1,227,385	1,225,557	99.88
58	PAP 733	1,123,907	1,122,508	99.88
59	PAP 739	1,141,361	1,139,632	99.84
60	PAP 749	1,363,536	1,361,513	99.87
61	PAP 761	941,915	935,642	99.83
62	PAP 764	883,071	881,568	99.86
63	PAP 765	893,214	892,046	99.86
64	PAP 766	954,639	953,144	99.87
65	PAP 767	775,540	774,621	99.86
66	PAP 773	797,495	796,467	99.87
67	PAP 777	1,447,886	1,441,337	99.87
68	PAP 784	1,056,203	1,054,466	99.88
69	PAP 790	1,120,950	1,119,496	99.71
70	PAP 792	944,459	942,565	99.88
71	PAP 795	943,086	941,925	99.86
72	PAP 796	1,042,448	1,041,158	97.51
73	PAP 815	1,174,597	1,170,622	99.83
74	PAP 814	751,683	750,690	99.84
75	PAP 816	963,468	962,008	99.84
76	PAP 823	1,082,911	1,081,699	99.84
77	PAP 830	1,037,429	1,036,061	99.87
78	PAP 835	834,364	833,384	99.86
79	PAP 845	1,224,644	1,220,282	99.87
80	PAP 932	1,178,108	1,176,590	99.90
81	PAP 1025	1,028,666	1,027,042	99.89
82	PAP 1062	1,000,886	999,698	99.88
83	PAP 1090	1,185,619	1,184,141	99.88
84	PAP 1129	1,148,524	1,146,997	99.89
85	PAP 1142	992,301	989,360	99.86
86	PAP 1168	803,204	802,144	99.89
87	PAP 1185	772,148	771,234	99.89
88	PAP 1224	888,063	887,026	99.88
89	PAP 180 A	718,216	717,236	99.86
90	PAP 180 B	1,049,972	1,048,685	99.86
91	PAP 229 A	1,154,420	1,151,341	97.62

Table S4. Comparison of the number of single nucleotide polymorphisms (SNPs) identified by the reference- and nonreference-based GBS pipelines for SNP calling using 91 opium poppy accessions.

	**Number of SNPs called**	
**-r**	**Reference-free (*de novo*)**	**Reference-based**
0	224,940	513,165
0.5	77,820	380,676
0.6	47,836	339,763
0.7	21,328	293,910
0.8	7,650	244,382
0.9	1,835	165,363

-r, minimum percentage of individuals in a population required to process a locus for that population. Both analyses were conducted in STACKS.

Table S5. Potential effects of the single nucleotide polymorphisms (SNPs) identified by GBS on the *P. somniferum* genome (annotation GCA_003573695.1_ASM357369v1) analysed using SnpEff v4.11.

**Number of effects**	**165,786**	**100.00%**
Genic regions	16,250	9.80%
Exon	5,365	3.24%
Intron	7,546	4.55%
5’ UTR	1,236	0.75%
Splice site donor	26	0.02%
Splice site region	346	0.21%
Splice site acceptor	20	0.01%
Transcript	8	0.01%
3’ UTR	1,703	1.03%
Intergenic regions	149,536	90.20%

Table S6. Summary of seed colour of the *Papaver* accessions included in this study. The RGB values were estimated from raw pictures using ColorChecker (X-rite) in Adobe Lightroom Classic CC software. The RGB values were converred to Hex colour codes with RGB Color code chart, and the corresponsding approximate colours were determined from Hex colour codes using HTML CSS Color Picker.

No.	Accession ID	RGB value			HEX colour code	Approximate colour
		R	G	B		
1	PAP 8	109	98	85	#6D6255	Makara
2	PAP 12	58	55	58	#3A373A	Fuscous Grey
3	PAP 28	74	67	65	#4A4341	Crater Brown
4	PAP 56	54	53	56	#363538	Martinique
5	PAP 59	162	144	112	#A29070	Pale Oyster
6	PAP 71	120	121	122	#787979	Rolling Stone
7	PAP 77	88	80	69	#585045	Mondo
8	PAP 78	56	59	68	#383B44	Vulcan
9	PAP 90	60	62	67	#3C3E43	Vulcan
10	PAP 107	109	104	102	#6D6866	Russett
11	PAP 133	46	48	55	#2E3037	Ebony
12	PAP 140	166	152	127	#A6987F	Bronco
13	PAP 142	92	75	68	#5C4B44	Saddle
14	PAP 146	56	58	62	#383A3E	Vulcan
15	PAP 149	53	53	55	#353537	Revolver
16	PAP 150	97	82	62	#61523E	Judge Grey
17	PAP 151	169	151	125	#A9977D	Bronco
18	PAP 152	164	147	116	#A49374	Pale Oyster
19	PAP 156	61	58	63	#353537	Revolver
20	PAP 160	102	103	103	#666767	Nevada
21	PAP 165	66	68	70	#424446	Arsenic
22	PAP 167	62	64	73	#3E4049	Blue Zodiac
23	PAP 197	109	97	97	#6D6161	Zambezi
24	PAP 200	172	160	135	#ACA087	Napa
25	PAP 201	121	116	112	#797470	Sand Dune
26	PAP 238	59	62	68	#3B3E44	Vulcan
27	PAP 246	46	43	41	#2E2B29	Bokara Grey
28	PAP 255	27	26	27	#1B1A1B	Mardi Gras
29	PAP 271	61	63	68	#3D3F44	Vulcan
30	PAP 281	63	66	72	#3F4248	Steel Grey
31	PAP 294	97	90	79	#615A4F	Makara
32	PAP 299	67	62	60	#433E3C	Paco
33	PAP 327	61	61	66	#3D3D42	Payne's Grey
34	PAP 333	30	27	26	#1E1B1A	Wood Bark
35	PAP 335	120	116	114	#787472	Hurricane
36	PAP 336	32	29	25	#201D19	Bokara Grey
37	PAP 337	37	32	27	#25201B	Bokara Grey
38	PAP 354	43	36	28	#2B241C	Black Magic
39	PAP 393	129	123	117	#817B75	Americano
40	PAP 400	43	38	36	#2B2624	Wood Bark
41	PAP 452	33	30	27	#211E1B	Bokara Grey
42	PAP 466	139	120	85	#8B7855	Cement
43	PAP 469	41	35	30	#29231E	Bokara Grey
44	PAP 478	40	37	36	#282524	Wood Bark

45	PAP 483	46	39	28	#2E271C	Black Magic
46	PAP 496	35	30	26	#231E1A	Bokara Grey
47	PAP 499	103	104	103	#676867	Finlandia
48	PAP 518	36	31	28	#241F1C	Bokara Grey
49	PAP 599	102	102	108	#66666C	Comet
50	PAP 612	98	88	64	#625840	Judge Grey
51	PAP 630	121	116	110	#79746E	Sand Dune
52	PAP 683	41	38	39	#292627	Aubergine
53	PAP 692	56	57	64	#383940	Payne's Grey
54	PAP 696	33	29	24	#211D18	Bokara Grey
55	PAP 715	48	40	34	#302822	Cocoa Brown
56	PAP 719	59	58	57	#3B3A39	Kilamanjaro
57	PAP 720	55	55	54	#373736	El Paso
58	PAP 727	109	102	100	#6D6664	Russett
59	PAP 733	51	50	57	#333239	Revolver
60	PAP 739	38	34	29	#26221D	Bokara Grey
61	PAP 747	33	30	26	#211E1A	Bokara Grey
62	PAP 749	116	99	91	#74635B	Dorado
63	PAP 761	169	153	125	#A9997D	Bronco
64	PAP 764	110	104	100	#6E6864	Dorado
65	PAP 765	87	80	67	#575043	Mondo
66	PAP 766	83	77	62	#534D3E	Panda
67	PAP 767	87	79	61	#574F3D	Panda
68	PAP 768	159	137	112	#9F8970	Mongoose
69	PAP 773	59	63	72	#3B3F48	Cloud Burst
70	PAP 777	36	32	29	#24201D	Bokara Grey
71	PAP 784	119	118	119	#777677	Old Lavender
72	PAP 790	60	58	63	#3C3A3F	Martinique
73	PAP 792	114	114	114	#727272	Dim Gray
74	PAP 795	38	34	27	#26221B	Black Magic
75	PAP 796	32	29	28	#201D1C	Wood Bark
76	PAP 815	49	41	29	#31291D	Black Magic
77	PAP 814	43	36	27	#2B241B	Black Magic
78	PAP 816	39	36	29	#27241D	Maire
79	PAP 823	37	34	33	#252221	Wood Bark
80	PAP 827	57	53	52	#393534	Gondola
81	PAP 830	108	99	94	#6C635E	Dorado
82	PAP 835	41	39	37	#292725	Bokara Grey
83	PAP 845	36	32	28	#24201C	Bokara Grey
84	PAP 932	54	57	65	#363941	Vulcan
85	PAP 1025	100	89	78	#64594E	Domino
86	PAP 1062	98	83	66	#625342	Judge Grey
87	PAP 1090	94	90	91	#5E5A5B	Don Juan
88	PAP 1129	52	51	55	#343337	Haiti
89	PAP 1142	158	135	108	#9E876C	Sandal
90	PAP 1168	100	95	95	#645F5F	Zambezi
91	PAP 1185	121	120	123	#79787B	Topaz
92	PAP 1224	120	122	118	#787A76	Gunsmoke
93	PAP 180 A	141	116	72	#8D7448	Shadow
94	PAP 180 B	140	114	69	#8C7245	Shadow
95	PAP 229 A	34	30	31	#221E1F	Aubergine

(a)

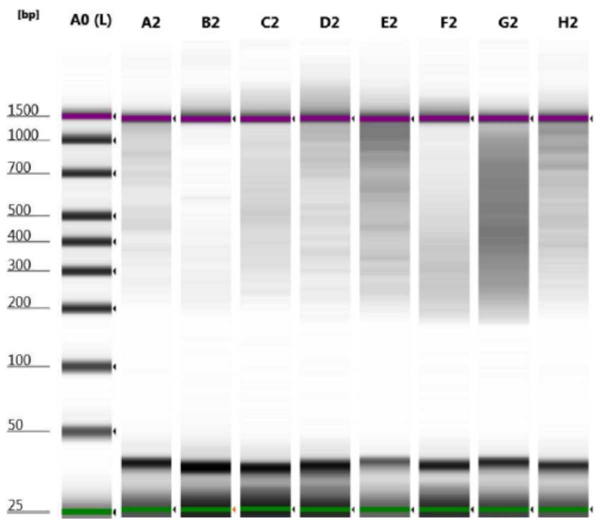

Gel Position	Enzyme Combination
**A2**	PstI/MspI
**B2**	PstI/MseI
**C2**	PstI/NlaIII
**D2**	PstI/HpyCH4IV
**E2**	EcoRI/MspI
**F2**	EcoRI/MseI
**G2**	EcoRI/NlaIII
**H2**	EcoRI/HpyCH4IV

(b) ***Pst*I/*Msp*I**

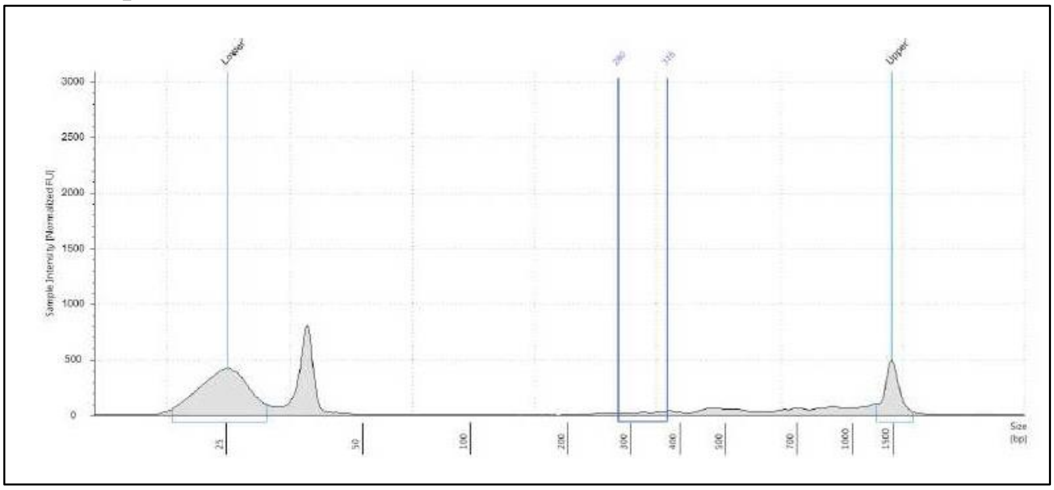

(c) ***Pst*I/*Mse*I**

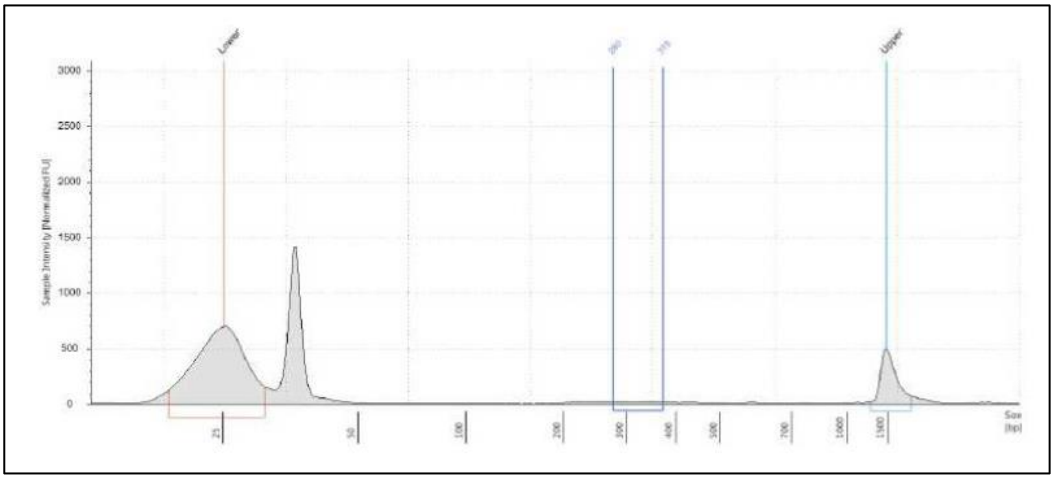

(Fig. **S1** continued)

(d) ***Pst*I/*Nla*III**

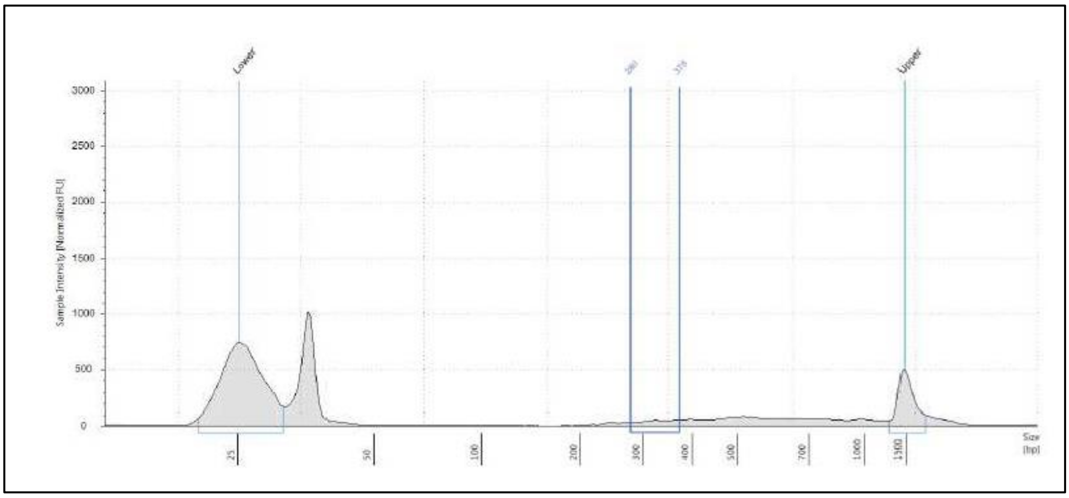

(e) ***Pst*I/*HpyCh4*IV**

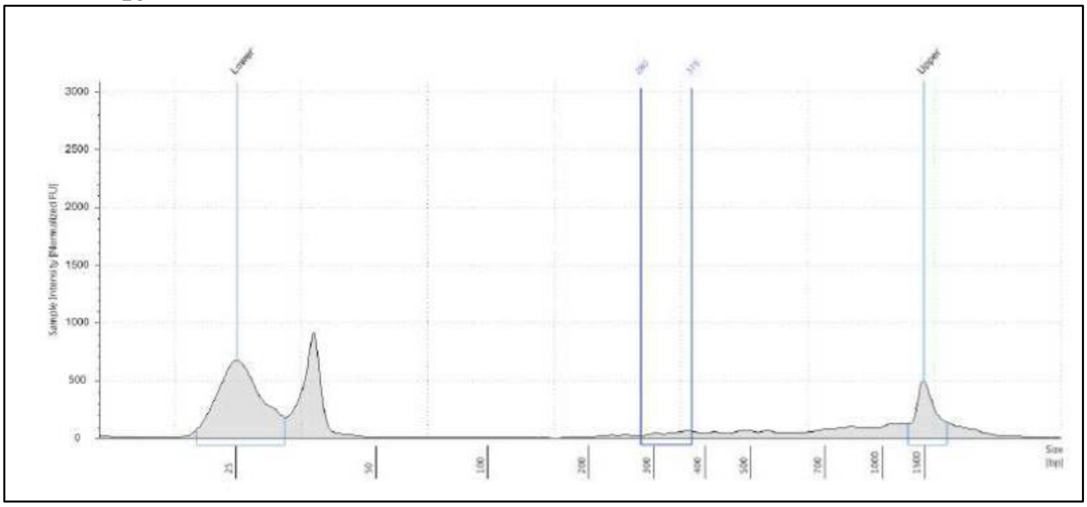

(f) ***Eco*RI/*Msp*I**

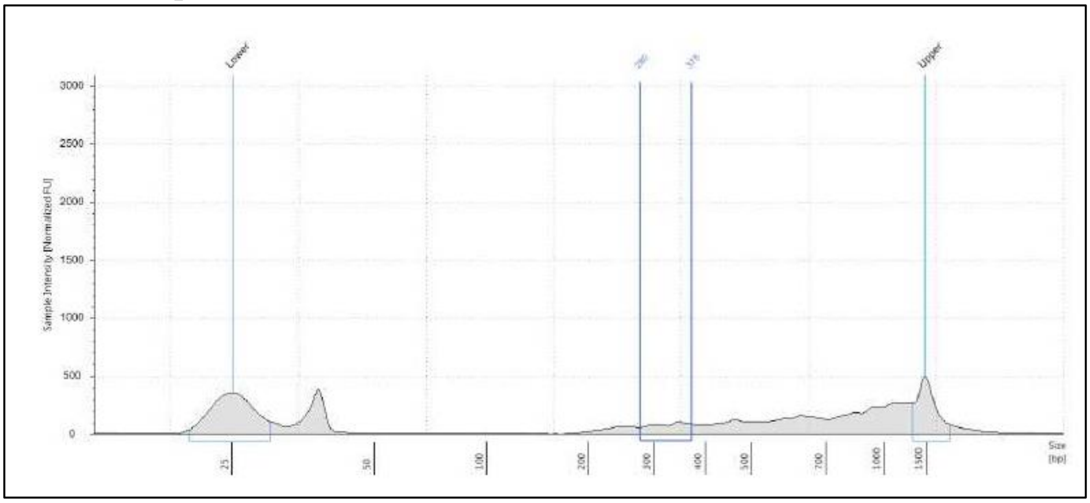

(Fig. **S1** continued)

(g) ***Eco*RI/*Mse*I**

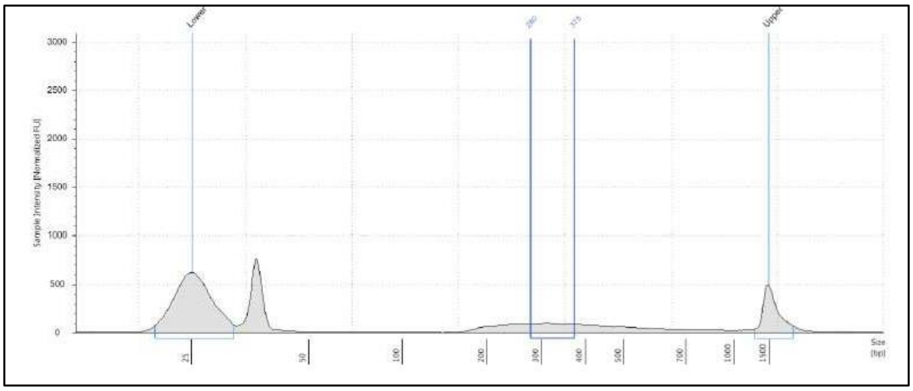

(h) ***Eco*RI/*Nla*III**

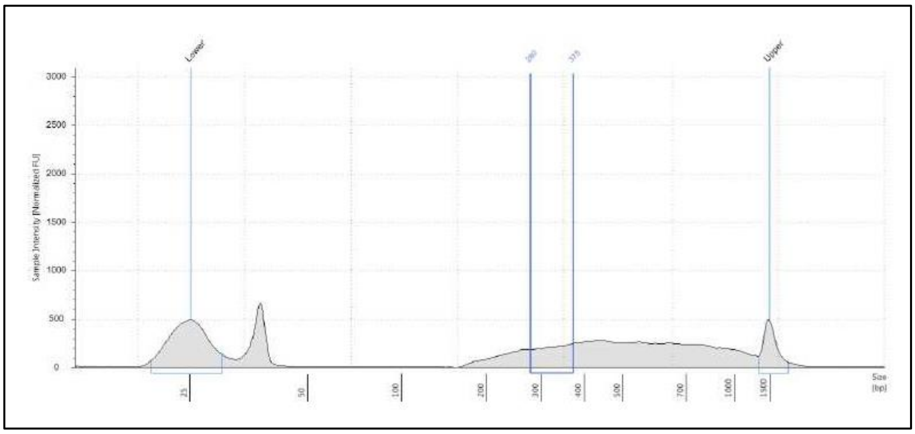

(i) *Eco*R*I*/*HpyCh4*IV

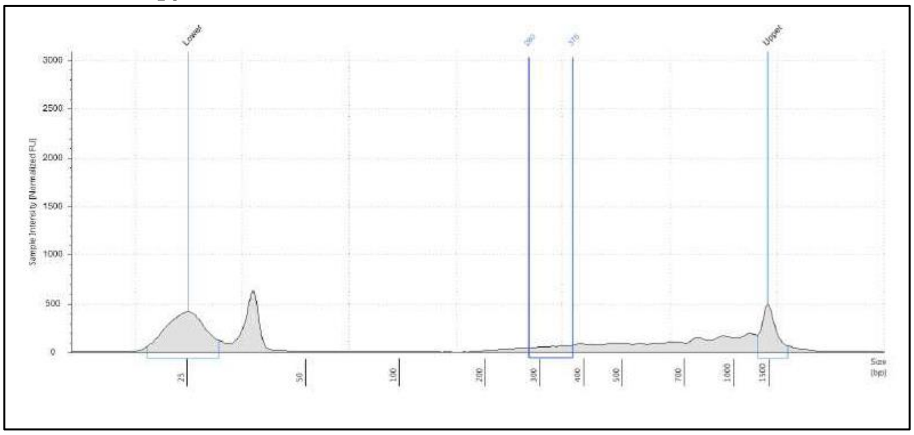

Fig. S1 Selecting the optimum restriction enzymes combination for GBS in *Papaver* species. (**a**) A gel view of the eighth double digested libraries from a pool of 3 representative samples used for selecting the optimal enzyme combination for GBS in *Papaver*. The eight enzyme combinations used are indicated to the right. (**b-i**) Electropherogram views of the eight double digested libraries from a pool of 3 representative samples used for selecting the optimal enzyme combination for GBS in *Papaver*. An even profile within the size selection window (280bp-375bp) means no or low repeat regions, which usually appear as a peak in the electropherogram view. The enzyme combination used for each electropherogram is show at the top.

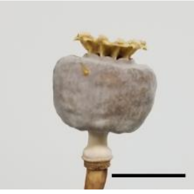
PAP 400
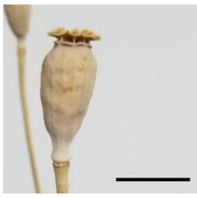
PAP 796
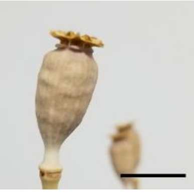
PAP 229 A
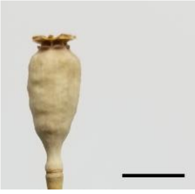
PAP 255
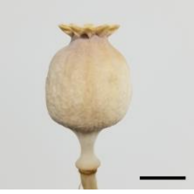
PAP 354
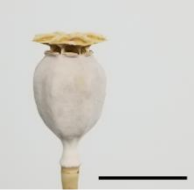
PAP 696
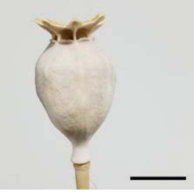
PAP 739
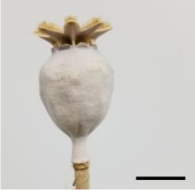
PAP 749
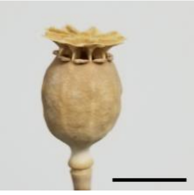
PAP 333
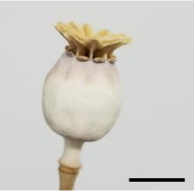
PAP 835
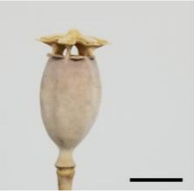
PAP 478
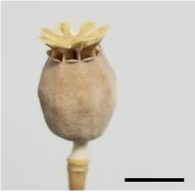
PAP 452
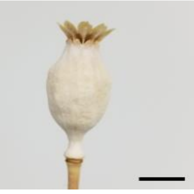
PAP 814
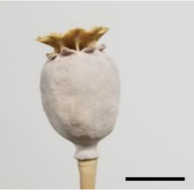
PAP 518
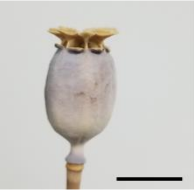
PAP 715
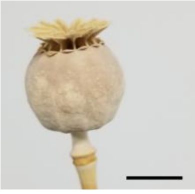
PAP 337
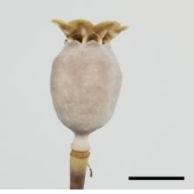
PAP 336
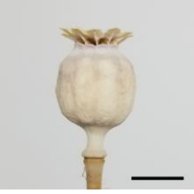
PAP 795
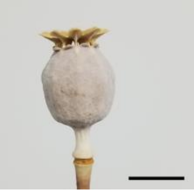
PAP 496
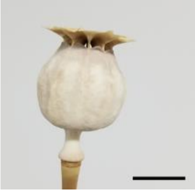
PAP 777
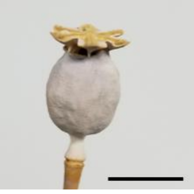
PAP 823
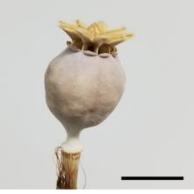
PAP 469
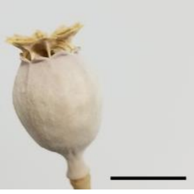
PAP 816
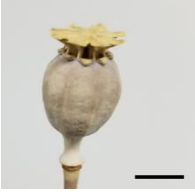
PAP 815
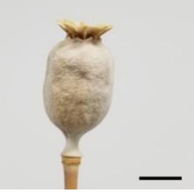
PAP 845
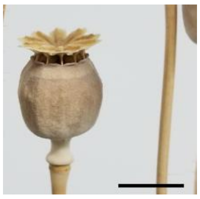
PAP 683
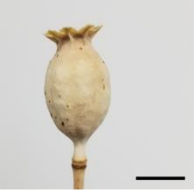
PAP 1142
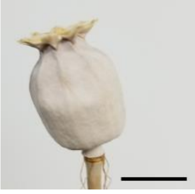
PAP 8
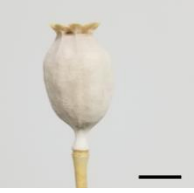
PAP 764
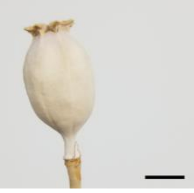
PAP 765
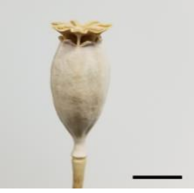
PAP 767
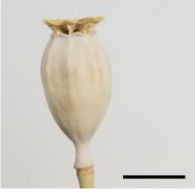
PAP 766

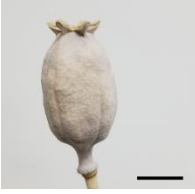
PAP 142
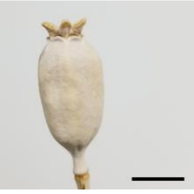
PAP 790
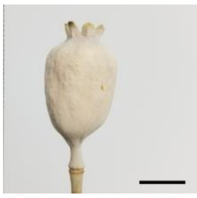
PAP 140
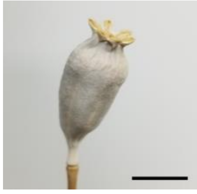
PAP 59
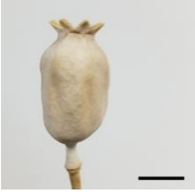
PAP 327
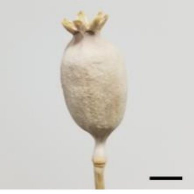
PAP 149
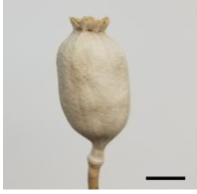
PAP 151
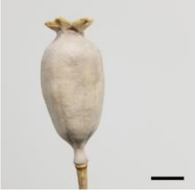
PAP 150
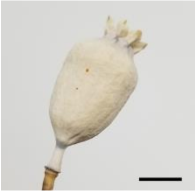
PAP 200
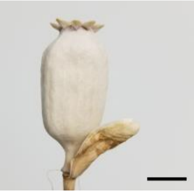
PAP 761
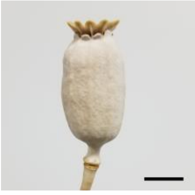
PAP 630
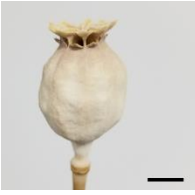
PAP 466
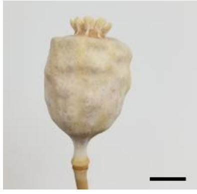
PAP 167
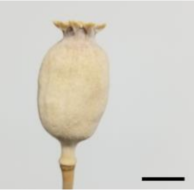
PAP 1090
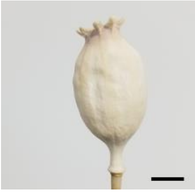
PAP 281
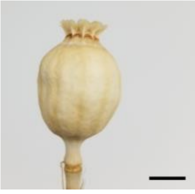
PAP 28
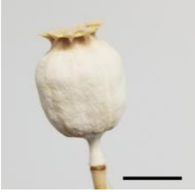
PAP 1224
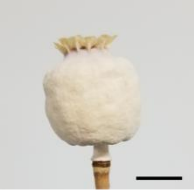
PAP 1185
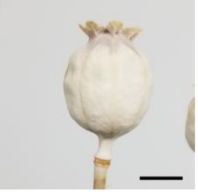
PAP 160
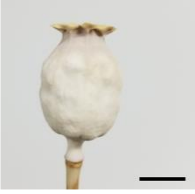
PAP 12
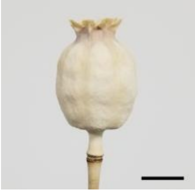
PAP 1129
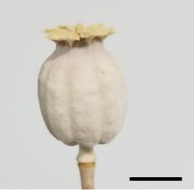
PAP 107
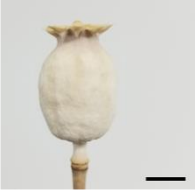
PAP 692
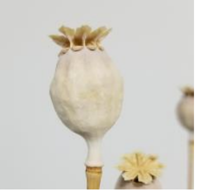
PAP 483
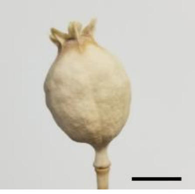
PAP 733
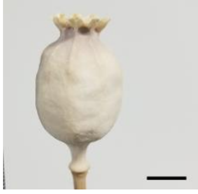
PAP 599
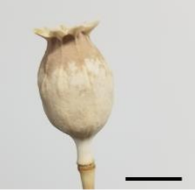
PAP 56
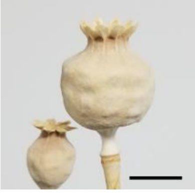
PAP 335
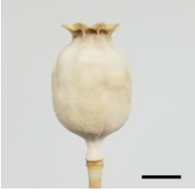
PAP 612
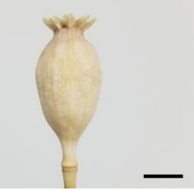
PAP 784
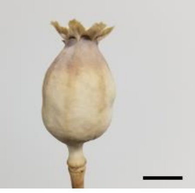
PAP 201
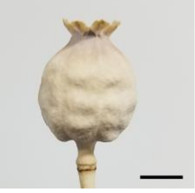
PAP 393

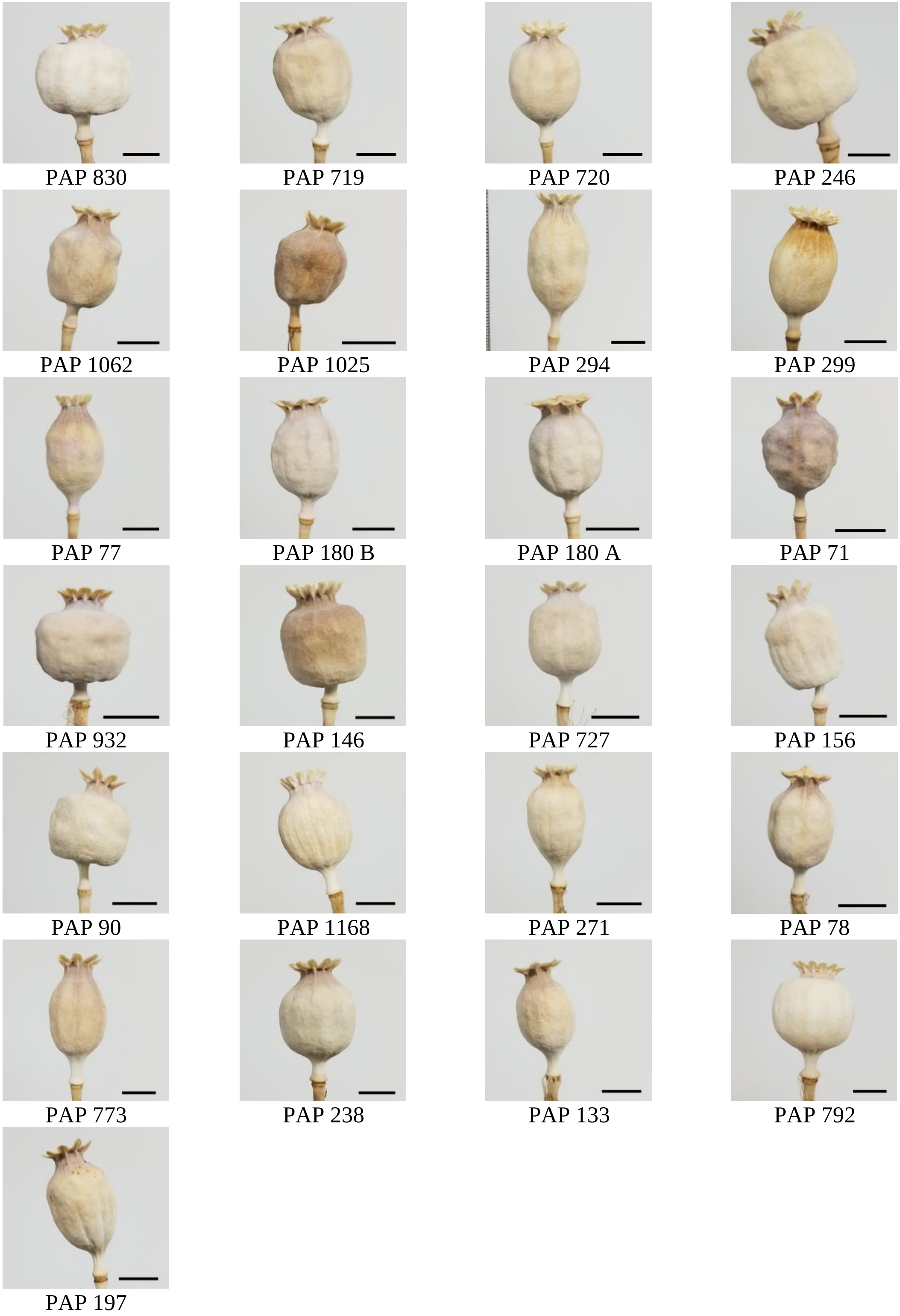

Fig. S2 Capsule morphology of the *Papaver* accessions included in this study. Bar = 1 cm. Images were taken using a SM-G950U1 digital camera under normal light indoors.

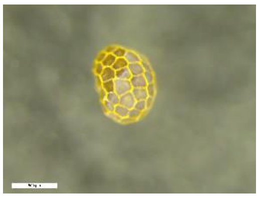
PAP 400

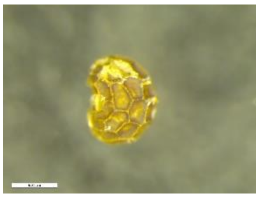
PAP 796

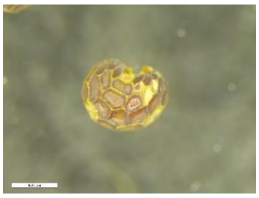
PAP 229 A

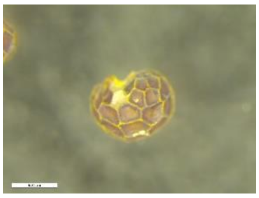
PAP 255

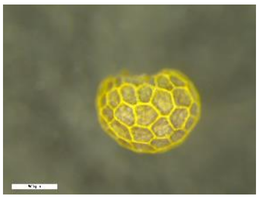
PAP 354

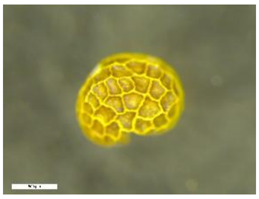
PAP 696

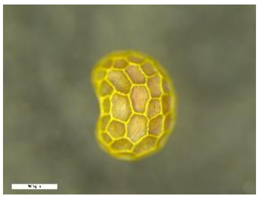
PAP 739

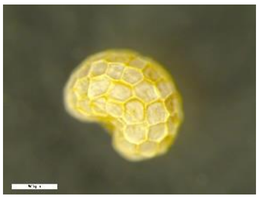
PAP 749

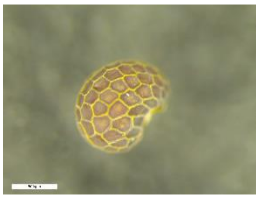
PAP 333

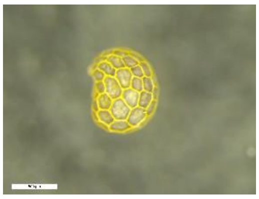
PAP 835

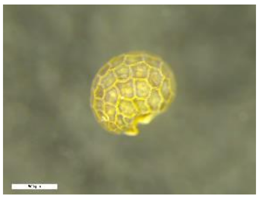
PAP 478

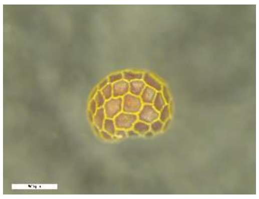
PAP 452

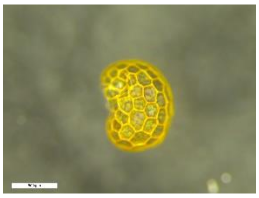
PAP 814

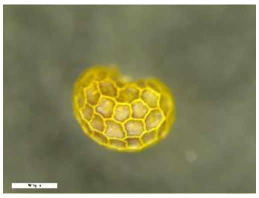
PAP 518

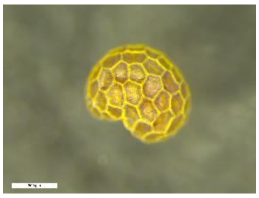
PAP 715

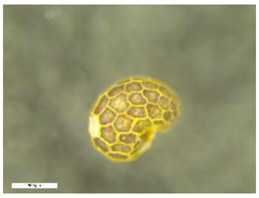
PAP 337

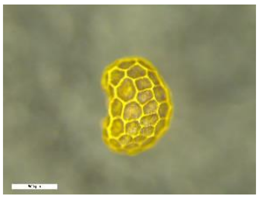
PAP 336

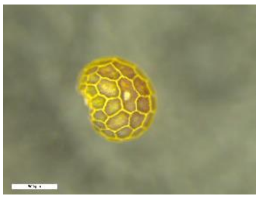
PAP 795

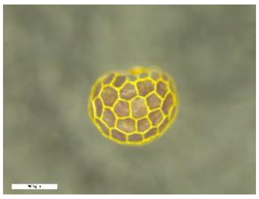
PAP 496

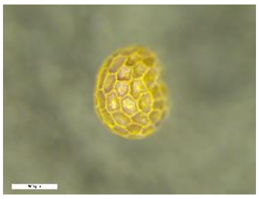
PAP 777

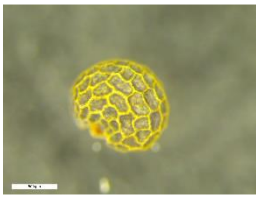
PAP 823

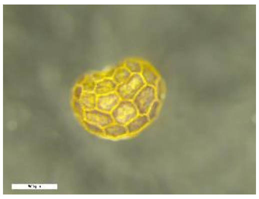
PAP 469

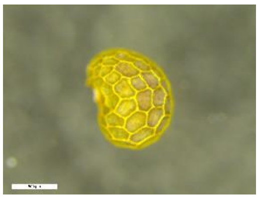
PAP 816

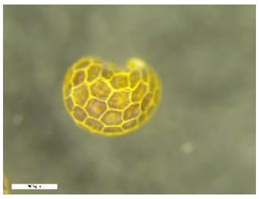
PAP 815

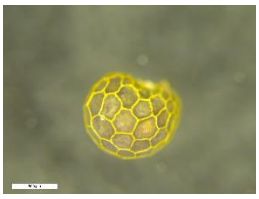
PAP 845

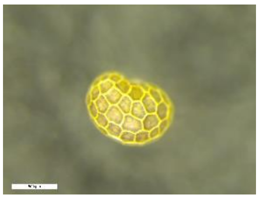
PAP 683

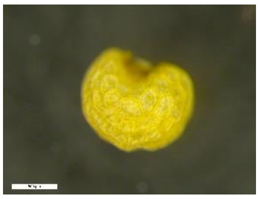
PAP 1142

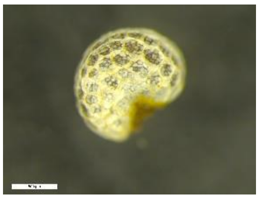
PAP 8

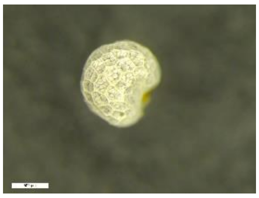
PAP 764

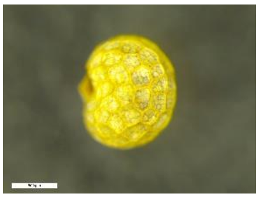
PAP 765

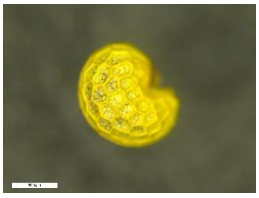
PAP 767

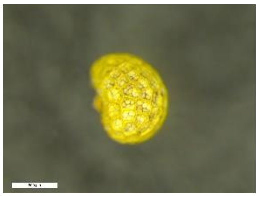
PAP 766

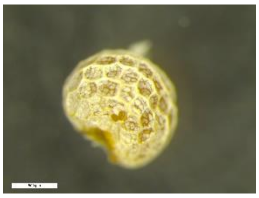
PAP 142
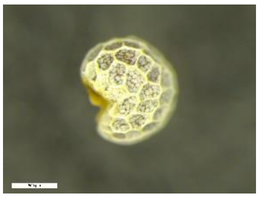
PAP 790
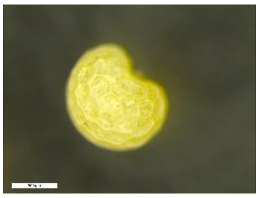
PAP 140
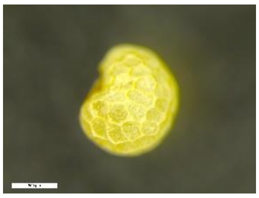
PAP 59
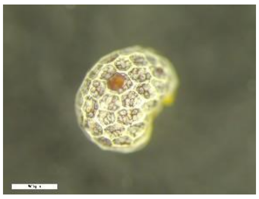
PAP 327
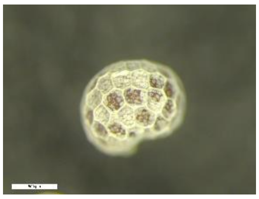
PAP 149
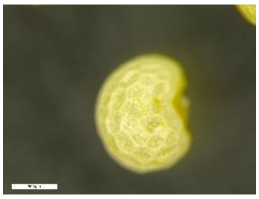
PAP 151
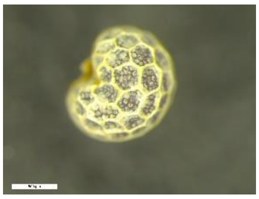
PAP 150
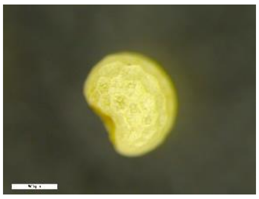
PAP 200
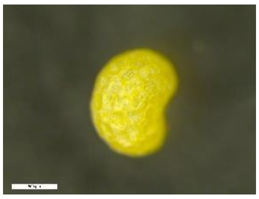
PAP 761
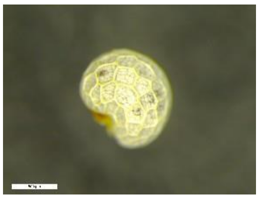
PAP 630
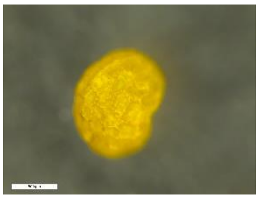
PAP 466
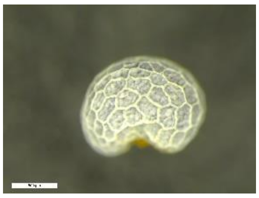
PAP 167
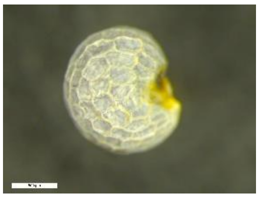
PAP 1090
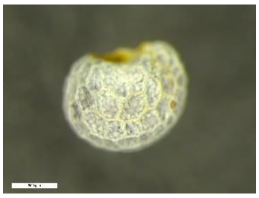
PAP 281
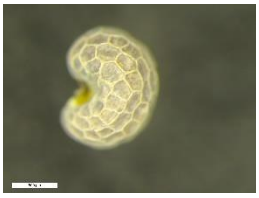
PAP 28
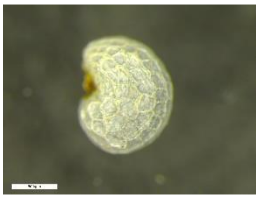
PAP 1224
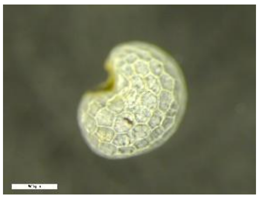
PAP 1185
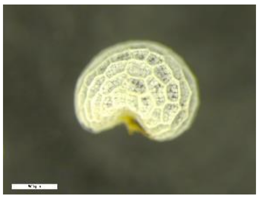
PAP 160
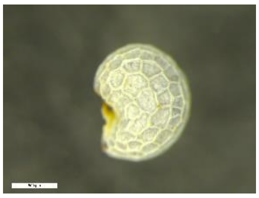
PAP 12
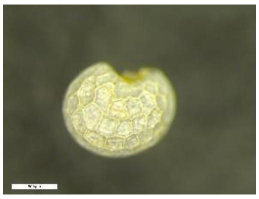
PAP 1129
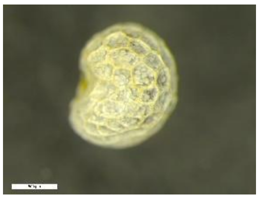
PAP 107
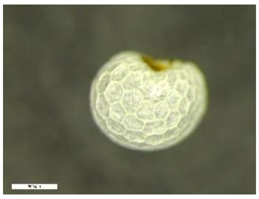
PAP 692
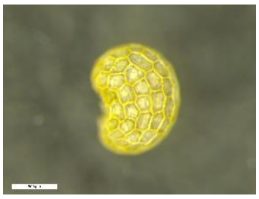
PAP 483
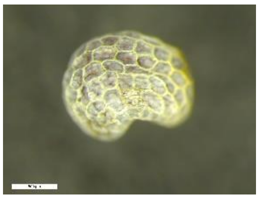
PAP 733
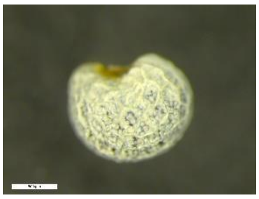
PAP 599
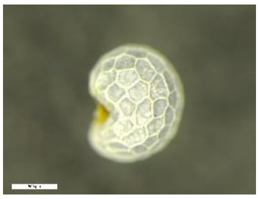
PAP 56
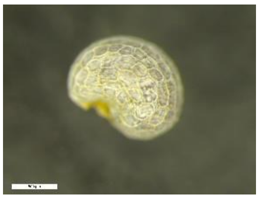
PAP 335
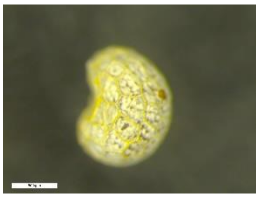
PAP 612
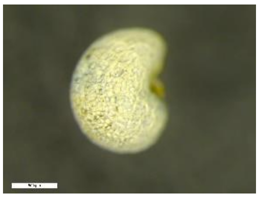
PAP 784
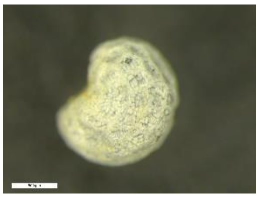
PAP 201
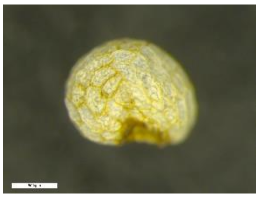
PAP 393

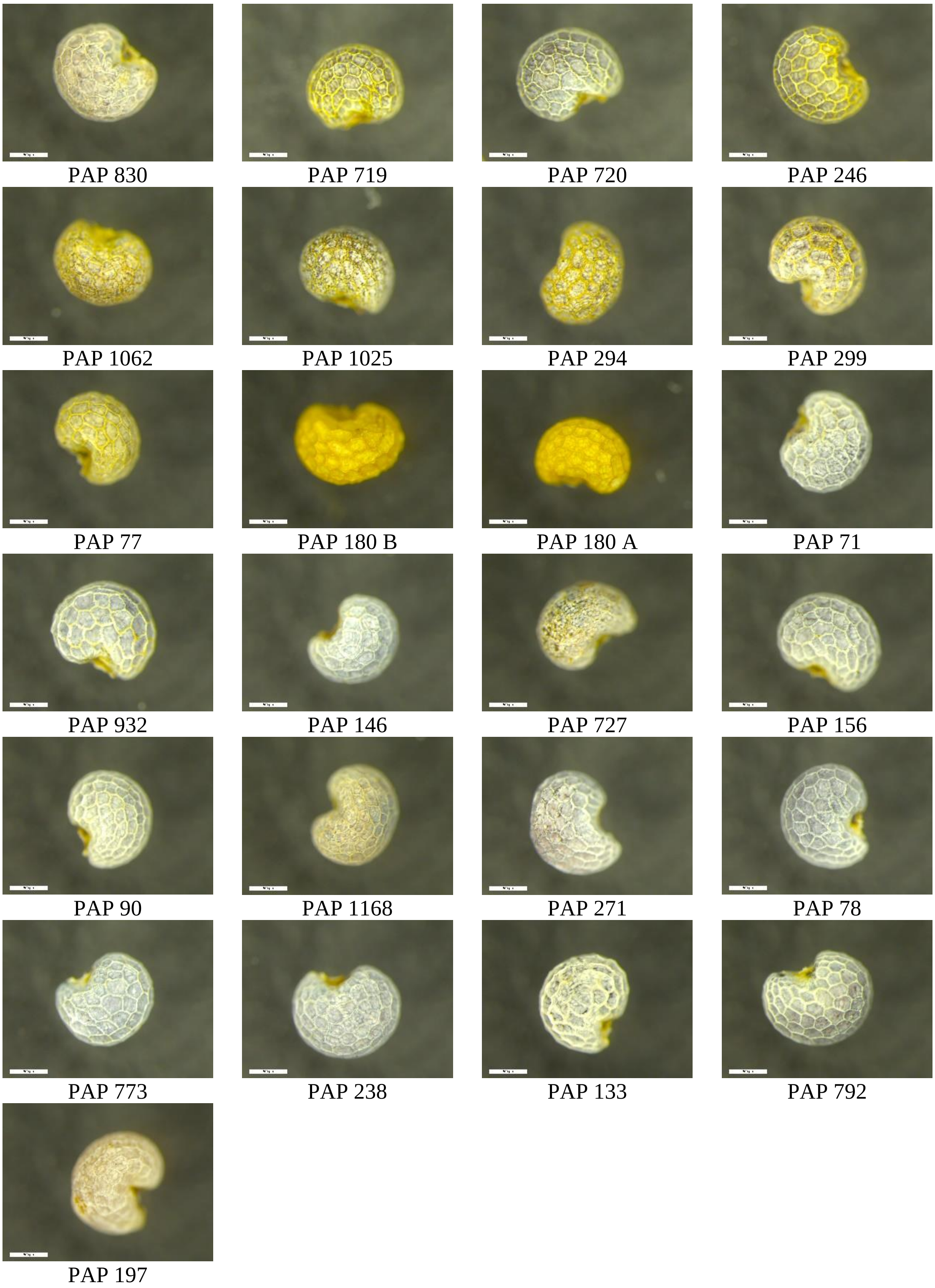

Fig. S3 Seed morphology of the *Papaver* accessions included in this study. Bar = 500 µm. Images were captured using a Leica M205 FA stereomicroscope with a digital camera Leica DFC 420 and Leica Application Suite Software (LAS 4.0, Leica) with 10% light and 50% zoom in condition.

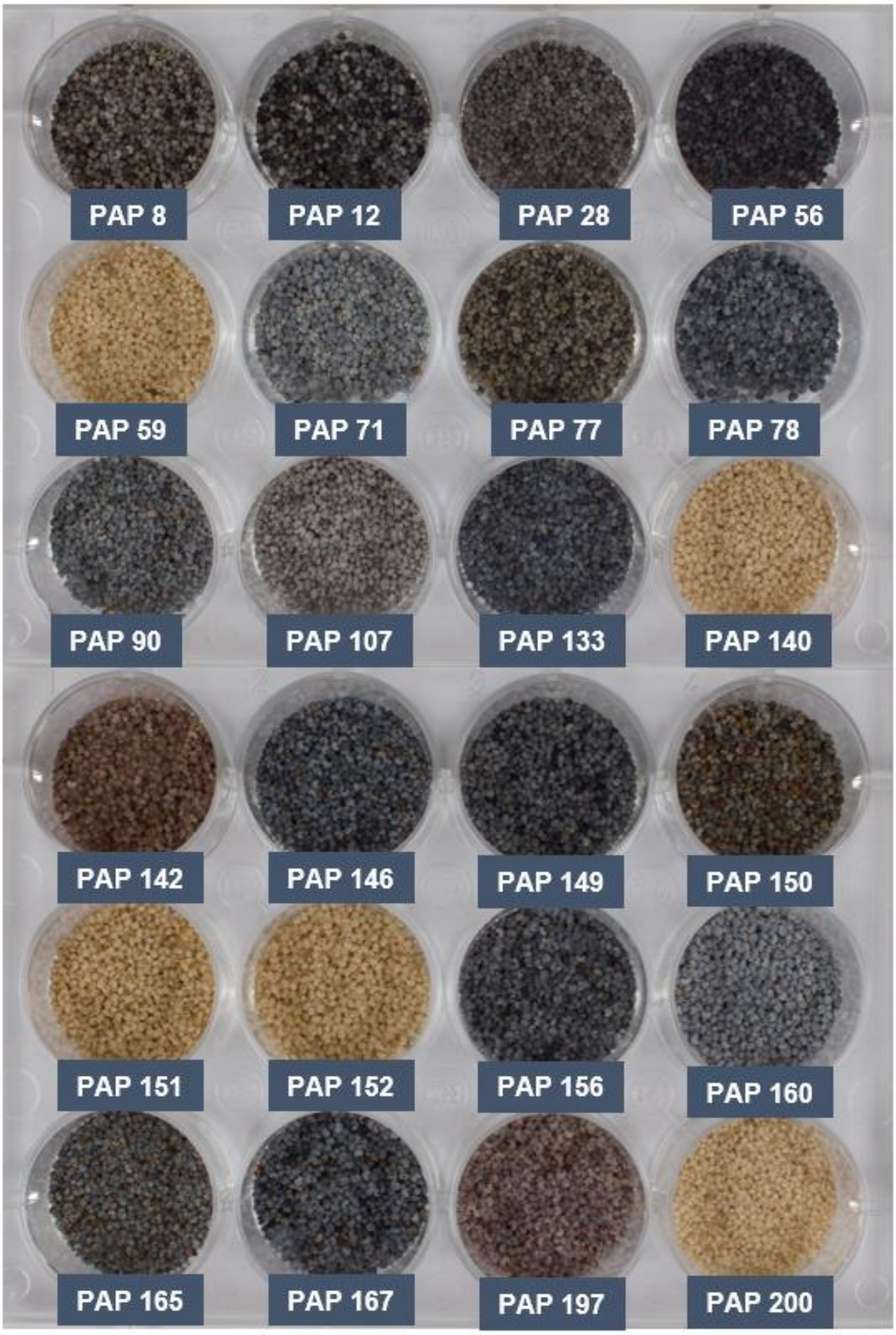

(Fig. **S4** continued)

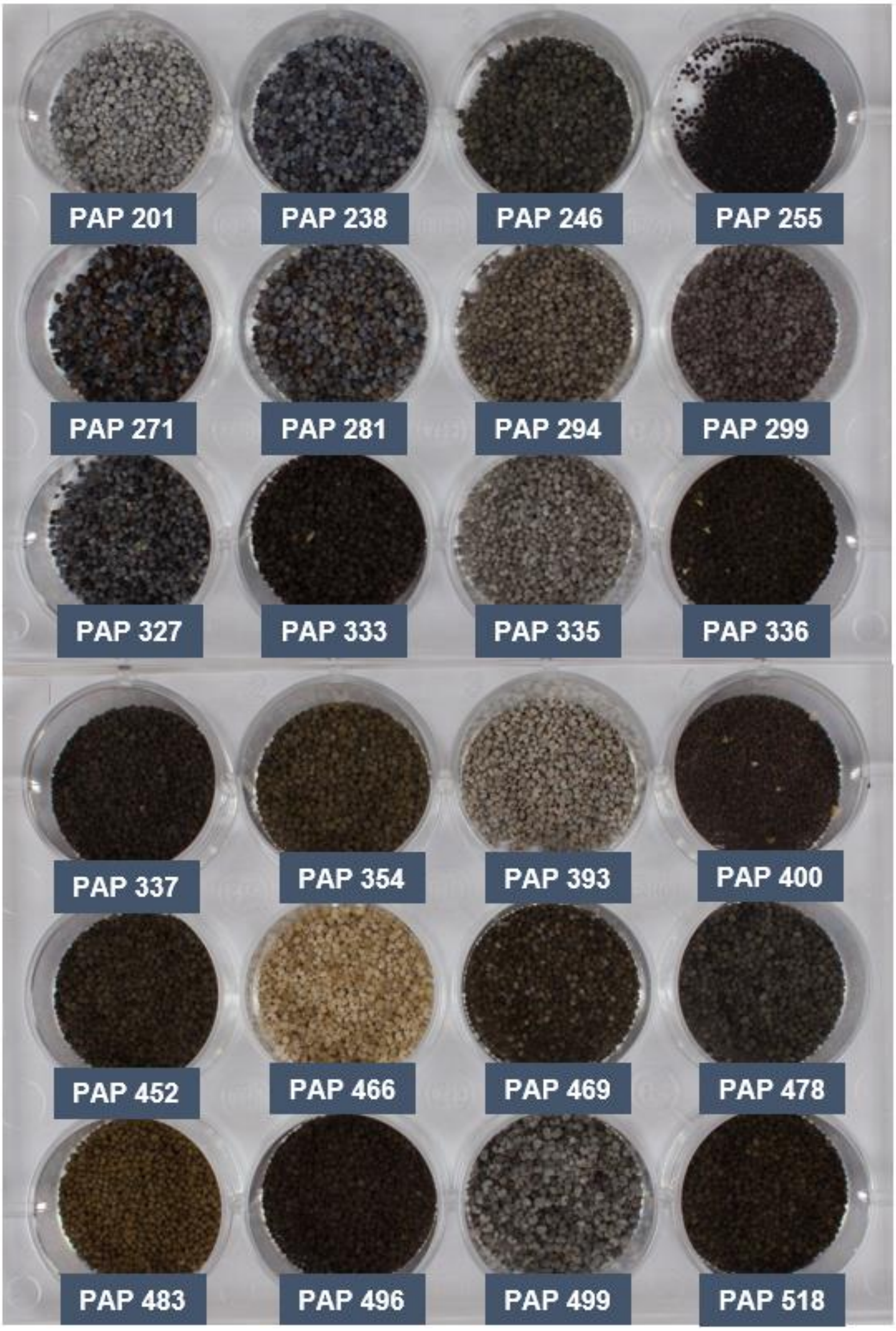

(Fig. **S4** continued)

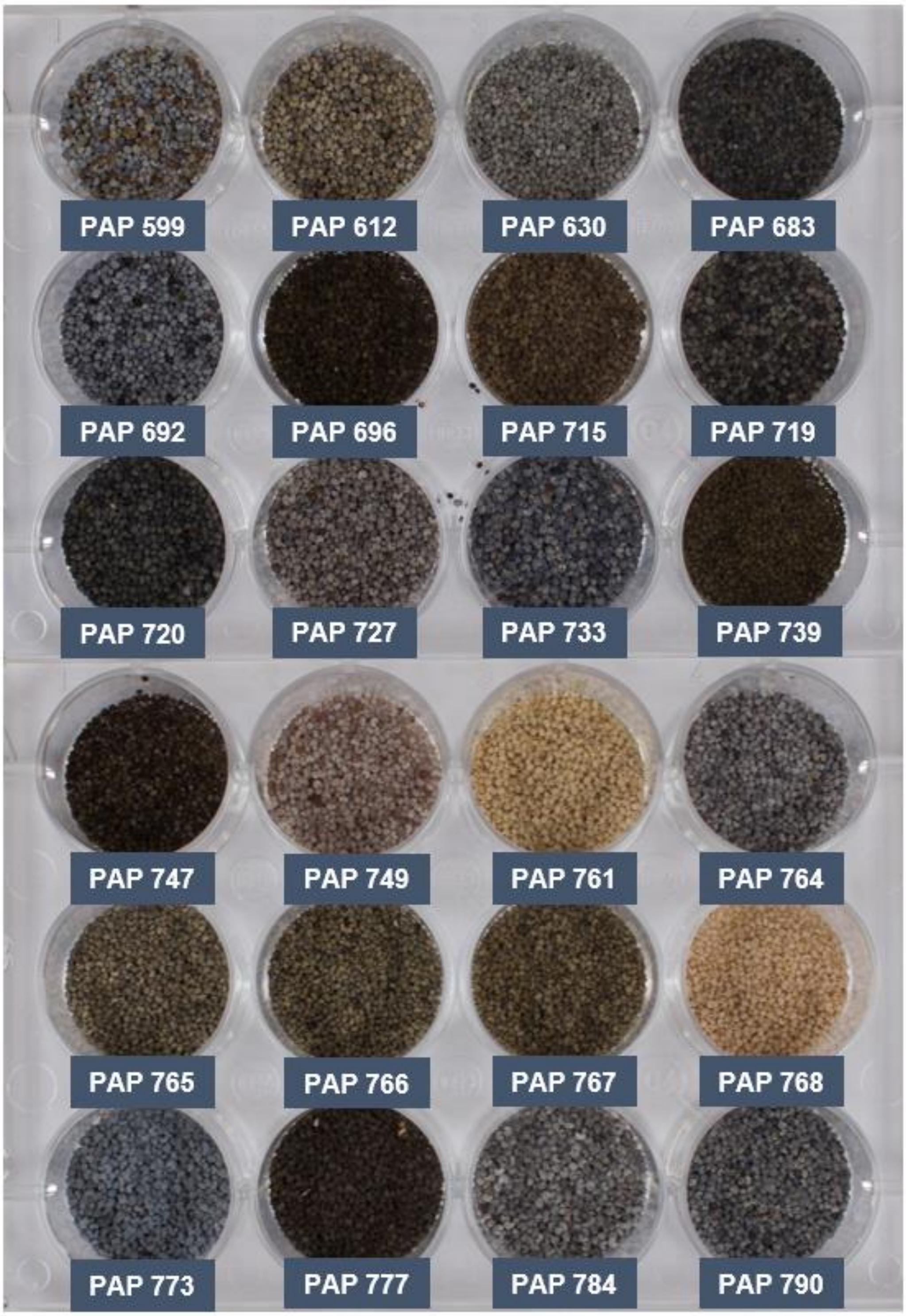

(Fig. **S4** continued)

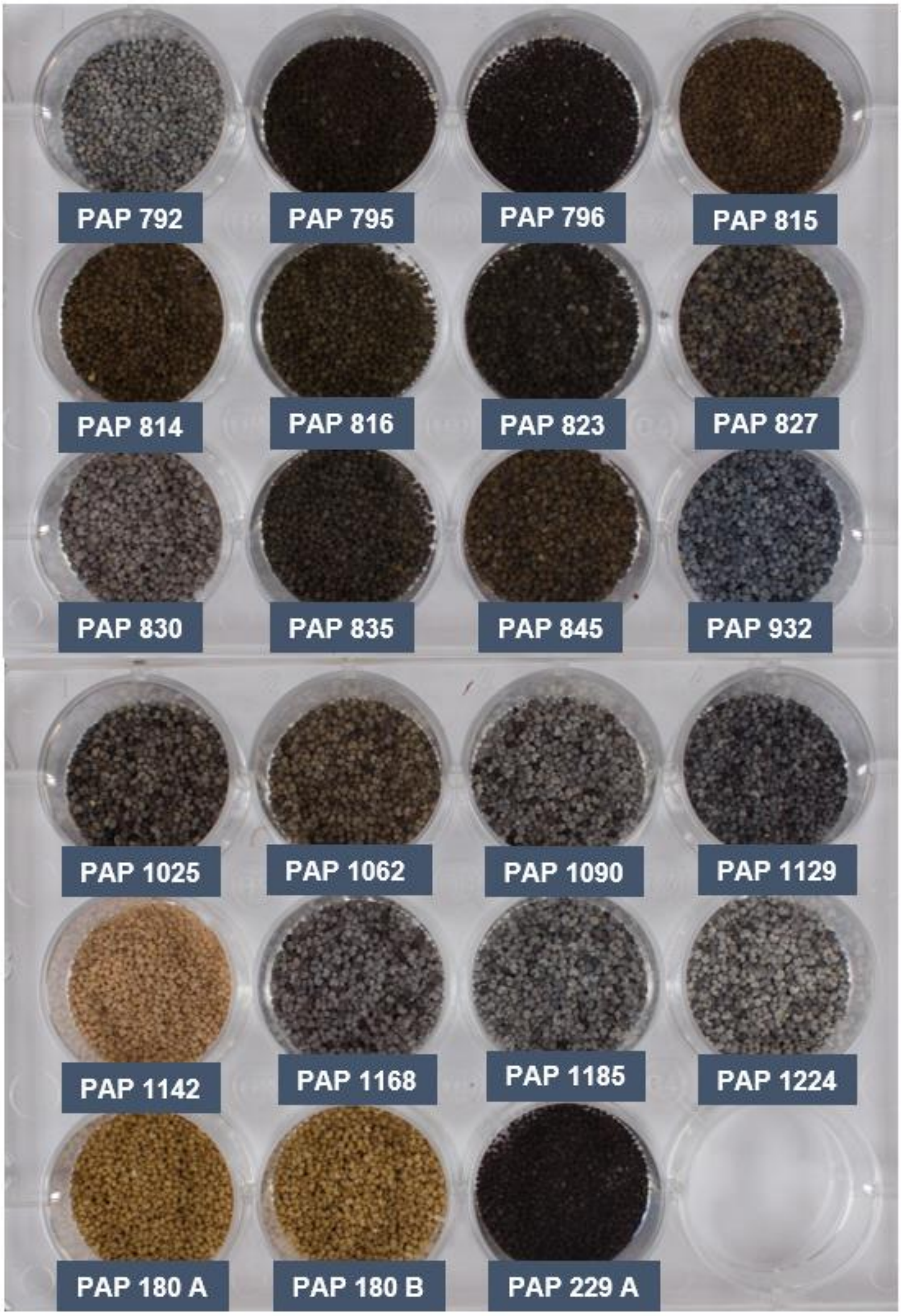

Fig. S4 Seed colour variability of the *Papaver* accessions included in this study. The RGB values were estimated from raw pictures using ColorChecker (X-rite) in Adobe Lightroom Classic CC software. The RGB values were converred to Hex colour codes, from which the corresponsding approximate colour were determined using HTML CSS Color Picker (see **Table S6**).

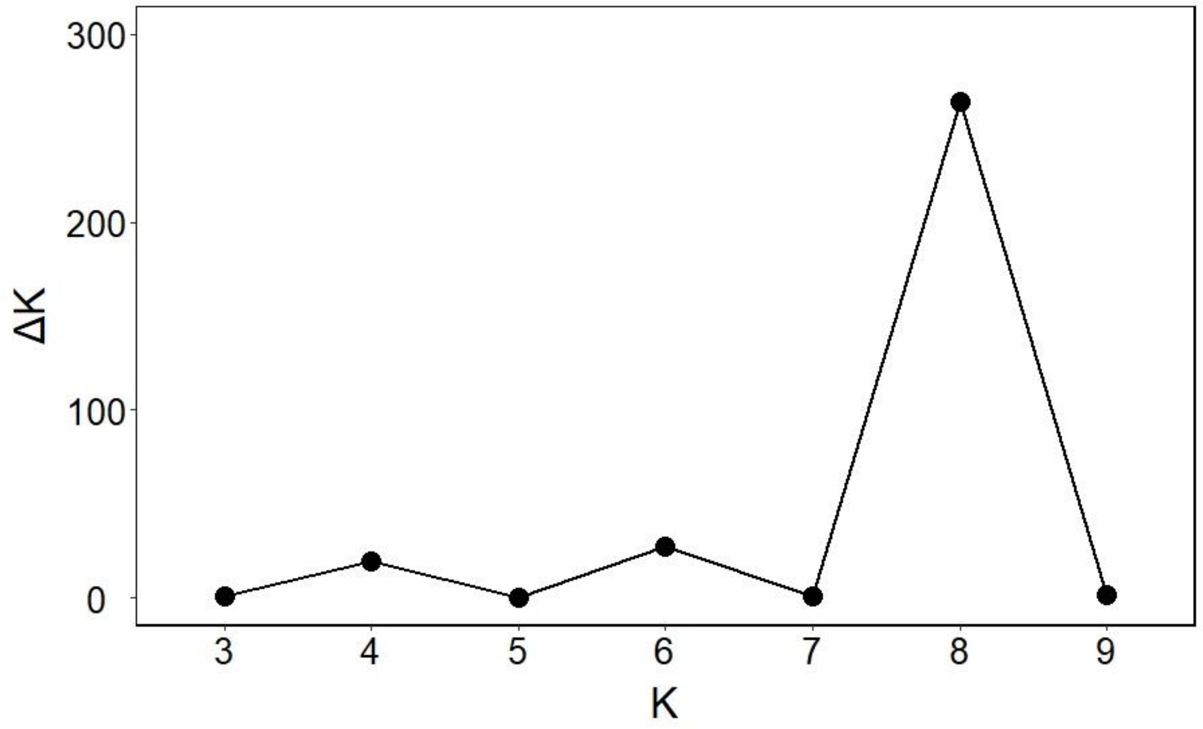

Fig. S5 Plot of ΔK values with K from 3 to 9 obtained from the STRUCTURE analysis.

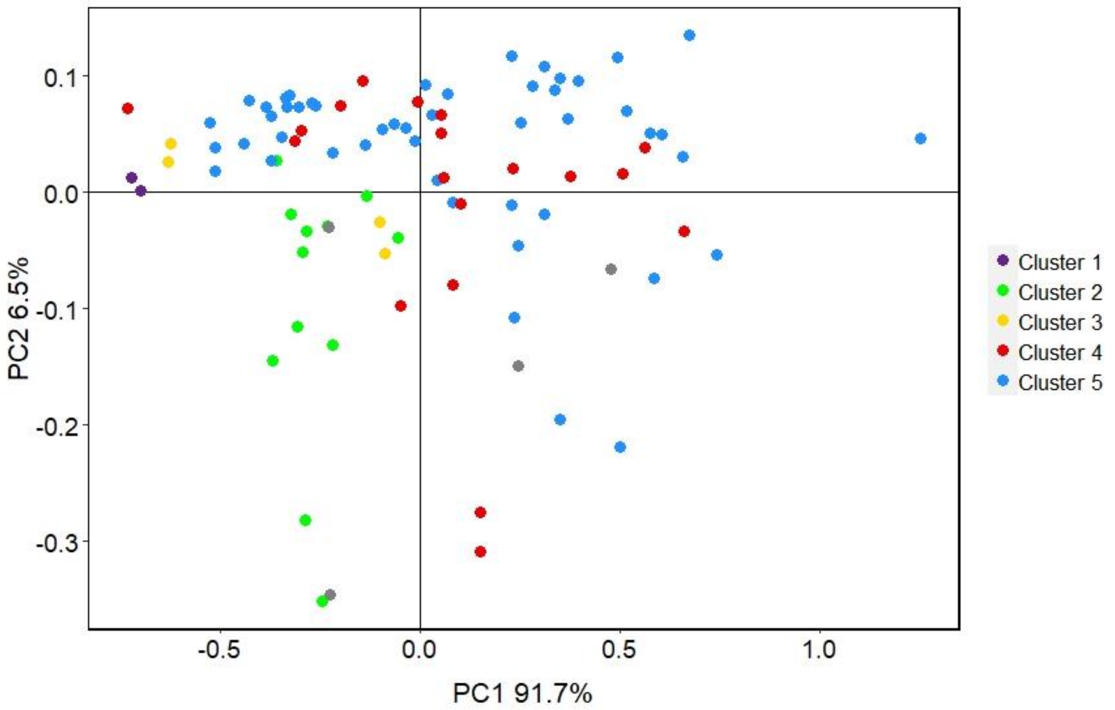

Fig. S6 A two-dimensional PCA score plot showing the diversity in alkaloid content. Axes are labelled with the percentage of variation explained by the two PCs. Colours indicate the five subpopulations identified by DAPC (**Fig. 5**). The accessions for which no GBS data was available are indicated with grey dots.
